# FOLR1 increases sensitivity to cisplatin treatment in ovarian cancer cells

**DOI:** 10.1186/s13048-018-0387-y

**Published:** 2018-02-13

**Authors:** Ming-ju Huang, Wei Zhang, Qi Wang, Zhi-jun Yang, Sheng-bin Liao, Li Li

**Affiliations:** 1 Department of Gynecology Oncology, Tumor Hospital of Guangxi Medical University, Nanning, 530021 China; 2 Department of Gynecology, Chongqing Three Gorges Central Hospital, Wanzhou District of Chongqing, 404000 China

**Keywords:** Folate binding protein, Ovarian cancer, SKOV3 cells, Cisplatin, Apoptosis, Cell cycle, Multidrug resistance

## Abstract

**Background:**

Whether there is a mechanistic link between FOLR1 and response to cisplatin has not been extensively examined. In this study, we determine the expression of FOLR1 in ovarian cancer and examine if FOLR1 levels influence response to cisplatin.

**Results:**

(1) FOLR1 protein expression was lowest in normal ovarian tissue, higher in benign ovarian tumors, and highest in malignant tumors (*P* < 0.01). (2) FOLR1 expression was decreased in platinum drug-resistant ovarian tumors compared to sensitive tumors (*P* < 0.01). Consistent with this, FOLR1 expression in tumors progressing following cisplatin treatment was lower than levels in tumors in remission (*P* < 0.01). (3) FOLR1 was successfully overexpressed at both the mRNA and protein levels following transfection in SKOV3 cells. (4) SKOV3 cells with FOLR1 overexpression were the most sensitive to cisplatin treatment (IC50 = 3.60 μg/ml) and exhibited the highest inhibition rates in the presence of the drug (*P* < 0.05). (5) The rate of apoptosis of SKOV3 cells increased with cisplatin treatment in a dose- and time-dependent manner (*P* < 0.05). Cisplatin also induced S phase arrest in a concentration-dependent manner (*P* < 0.05). Apoptosis and S phase proportion were significantly altered by FOLR1 overexpression (*P* < 0.05).

**Conclusion:**

FOLR1 may be a useful biomarker for ovarian cancer, and it may be useful as a therapeutic application to improve sensitivity to cisplatin treatment.

## Background

Ovarian cancer is a serious malignancy, with high mortality and a five-year survival rate of approximately 20% - 30% for the prevailing advanced presentations [1]. Survival in patients with ovarian cancer can be improved with early detection, thorough surgery, and improved sensitivity to cisplatin-based chemotherapy. Folate binding protein (FOLR1) is a member of the human folate binding protein family. The gene is located on chromosome 11q13.3-14.1. FOLR1 is a glycosyl phosphatidylinositol connected membrane glycoprotein, consisting of 257 amino acids. The protein is completely exposed to extracellular molecules and anchored at the cell membrane by GPI [2]. FOLR1 is involved in DNA replication and damage repair. Its expression levels are closely related with tumor progression and cell proliferation [3, 4]. FOLR1, also known as folate receptor proteins, mediates cellular responses to folate, including cell division, proliferation, and tissue growth [5]. Few publications have reported on FOLR1 expression in ovarian tissue. Shen et al. found that FOLR1 expression was decreased in cisplatin-resistant tumors [6],but whether there is a mechanistic link between FOLR1 and response to cisplatin has not been extensively examined. In this study, we determine the expression of FOLR1 in ovarian cancer and examine if FOLR1 levels influence response to cisplatin. The data we provide here suggest that FOLR1 may be a useful predictive biomarker for cisplatin sensitivity in ovarian cancer.

## Results

### Expression of FOLR1 in normal ovary, benign ovarian tumors, and ovarian cancer

**Fig. 1:**
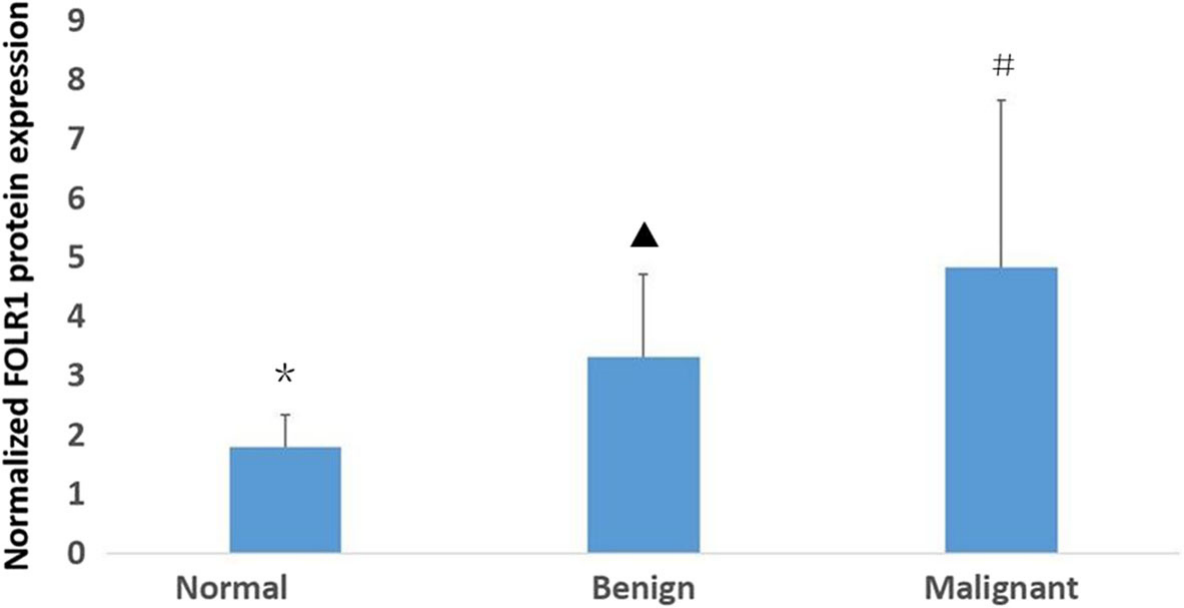
Ovarian tissues expression of FOLR1 protein detected by western blot.*,*P* = 0.000,compared with benign;▲,*P* = 0.002,compared with malignant;#,*P* = 0.000,compared with normal

Expression of FOLR1 in normal, benign, and cancerous ovarian tissues was determined by Western blot. GAPDH was used as a loading control. Expression of FOLR1 was lowest in normal ovarian tissue. FOLR1 was more highly expressed in benign tumors and even higher in malignant disease (*P* < 0.01)(Fig. 1).

### Expression of FOLR1 in cancerous ovarian tissue is correlated with clinicopathologic factors

**Fig. 2:**
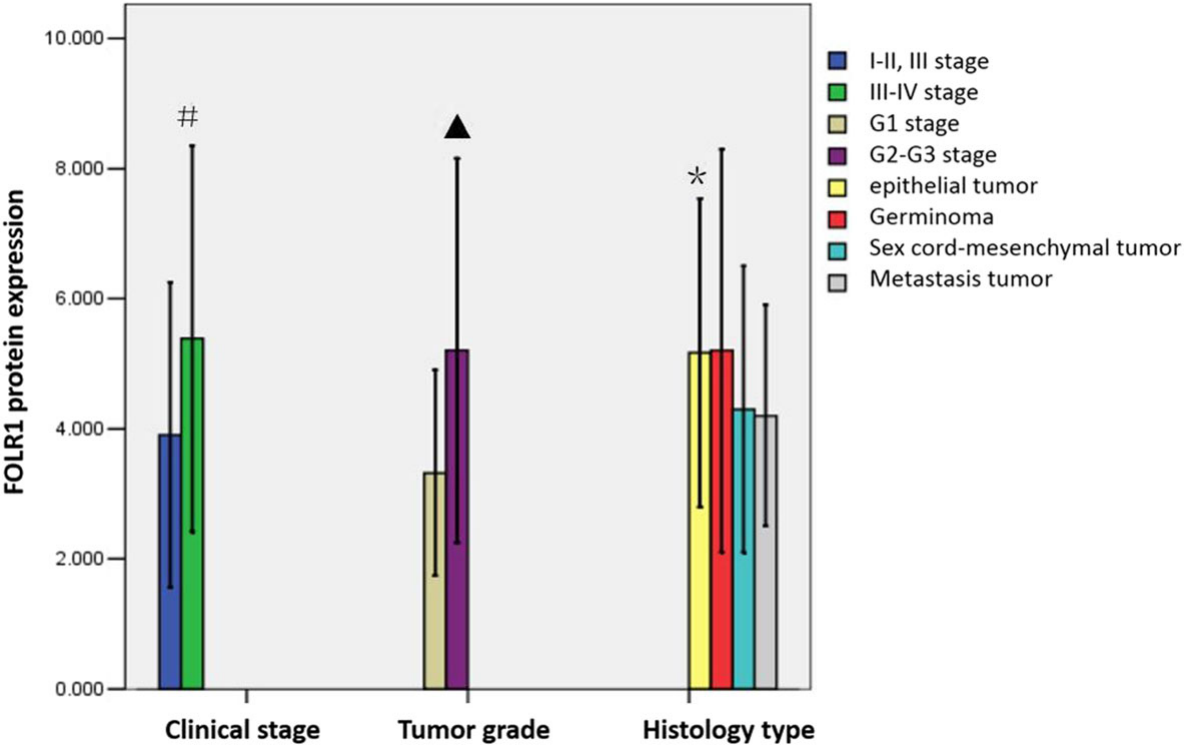
The comparison for expression of FOLR1 with different clinicopathological factors.#,*P* = 0.047,mucinous vs serous;▲,*P* = 0.013,G1 vs G2-G3;*,*P* = 0.013,stage I-II vs stage III-IV

FIOG (International Federation of Gynaecology and Obstetrics) system 2000 is used to determine the stages of malignant ovarian patients. The expression of FOLR1 in stage I-II is lower than that in stage III-IV and also lower in the well-differentiated than that in the low-differentiated (*P*<0.05). While there is no difference for expression of FOLR1 in the four different pathologic tissue, the significant difference does exist between the mucinous and the serous (*P*<0.05). The specific results are shown in Fig. 2.

### Correlation of expression of FOLR1 with tumor metastasis and ascites

**Fig. 3:**
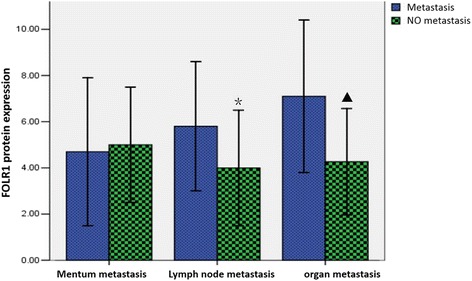
Comparison of expression of FOLR1 before and after metastasis.*,*P* = 0.01,compared with metastasis to lymph node;▲,*P* = 0.000,compared with metastasis to distant organ

Expression of FOLR1 in cancerous ovarian tissue with metastasizing to distant lymph node and/or organ is higher than that without metastasis (*P*<0.05).However,there’s no significance about correlation of expression of FORL1 with whether metastasizing to the greater omentum or having asites (*P* > 0.05). The specific results are shown in Fig. 3.

### FOLR1 expression in ovarian cancer tissue is correlated with patient treatment efficacy and drug resistance

**Fig. 4:**
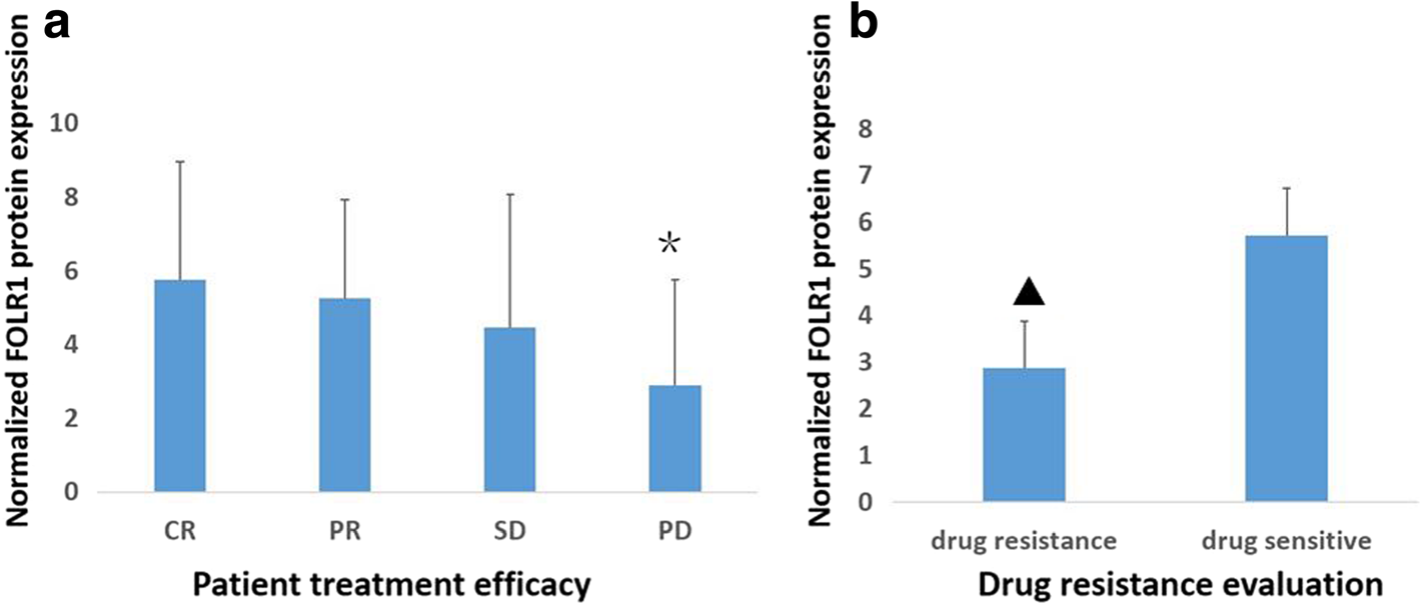
FOLR1 expression correlated with patient treatment efficacy (**a**) and drug resistance (**b**).*, *P* = 0.001, compared with CR + PR + SD; ▲, *P* = 0.000, compared with drug sensitive. CR, complete response. PR, partial response.SD, stable disease. PD, progressive disease

FOLR1 protein expression was highest in patients with complete remission (complete response, CR). FOLR1 expression decreased with decreased drug sensitivity (partial response, PR > stable disease, SD > progressive disease, PD). The difference in expression in CR, PR, and SD patients compared to PD patients was statistically significant (*P* < 0.01). The difference in expression in CR and PR patients compared to PD and SD patients was also significant (*P* < 0.05). However, there was no statistically significant difference in expression in CR and PR patients compared to SD patients (*P* > 0.05). Expression of FOLR1 in platinum drug-resistant ovarian cancer was lower than in platinum drug-sensitive tumors (*P* < 0.01),and after further chemotherapy expression of FOLR1 in PD was still lower than that in remission (*p* < 0.01) (Fig. 4).

### Correlation of expression of FOLR1 in ovarian cancer tissue with prognosis of patients

**Fig. 5:**
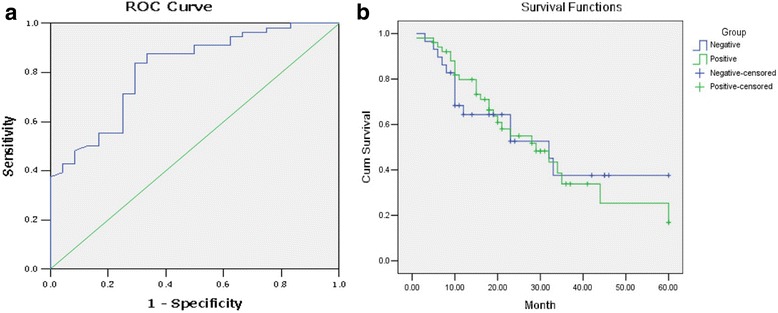
Expression of FOLR1 associated ROC curve. The maximum Youden index is 3.115 (**a**).Kaplan-Meier survival curve with cut-off value of expression of FOLR1 (**b**). The median OS is 29.4 m for the positive group (expression of FOLR> 3.115),and 32.3 m for the negative group (expression of FOLR< 3.115)

ROC curve that determines the relationship of FOLR-1 expression and nature of ovarian cancer demonstrates that maximum Youden index is 3.115. The specific result is shown in Fig. 5a.

Univariate analysis of Kaplan-Meier survival curve demonstrates that median overall survival time is 29.4 months for the positive group and 32.3 months for the negative group respectively, and the difference is not statistically significant (*P* > 0.05)(Fig. 5b).

COX multivariate analysis denies expression of FOLR-1 as an independent prognostic factor.

### FOLR1 expression in transfected SKOV3 cells

**Fig. 6:**
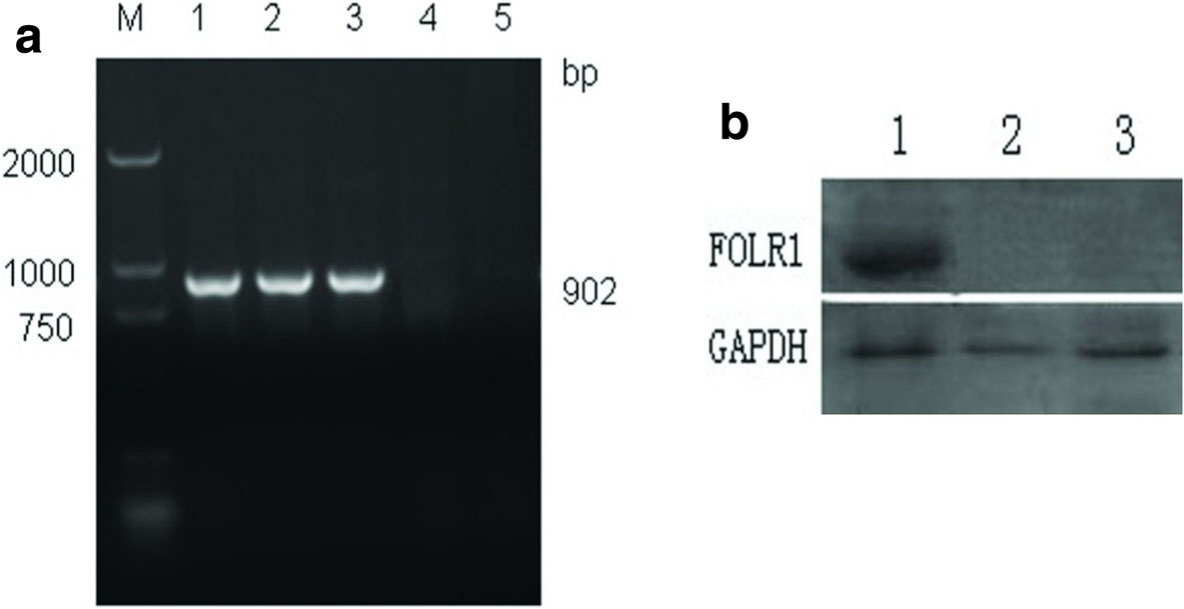
RT**-**PCR of FOLR1 in each of the different groups (**a**) (lane M, DL2000 marker; lanes 1-3, rpWPI-FOLR1-SKOV3 group; lane 4, pWPI-SKOV3 group; lane 5, SKOV3 group). Expression of FOLR1 protein in different groups detected by Western blot (**b**) (lanes 1, rpWPI-FOLR1-SKOV3 group; lane 2, pWPI-SKOV3 group; lane 3, SKOV3 group)

RT-PCR and Western blot were performed to confirm overexpression of FOLR1 at both the mRNA and protein levels in SKOV3 cells following transfection. Neither FOLR1 mRNA nor protein was detected in the empty vector transfected group (pWPI-SKOV3) or in control cells (SKOV3) (Fig. 6).

### Growth inhibition and apoptosis of SKOV3 cells treated with cisplatin

MTT assays were performed and IC_50_ values of each group were determined in the presence of cisplatin. The IC_50_ values of SKOV3, pWPI-SKOV3, pWPI-FOLR1-SKOV3 cells were 5.01, 4.96, and 3.60 μg/ml, respectively. The IC_50_ to cisplatin of the pWPI-FOLR1-SKOV3 group was significantly lower than the two control groups (*P* < 0.05). There was no significant difference in IC_50_ between the two control groups (*P* > 0.05). For all groups, the growth inhibition rate in the presence of cisplatin increased in both a time- and dose-dependent manner. Apoptosis of cells in the three groups increased with cisplatin treatment in a similar manner. Under the same conditions, pWPI-FOLR1-SKOV3 cells displayed the highest growth inhibition rate compared to the other two groups (*P* < 0.05). Apoptosis also increased in each of the three groups in a dose- and time-dependent manner. Under the same conditions, pWPI-FOLR1-SKOV3 cells displayed the highest rates of apoptosis compared to the other groups (*P* < 0.05). There was no significant different between the two control groups (*P* > 0.05). The specific results are shown in Table 1.

**Table 1:** Growth inhibition rate and apoptosis rate of each group of cells treated with different concentrations of cisplatin for different lengths of time

Group	Cases	Growth inhibition rate			Apoptosis rate		
		24 h	48 h	72 h	24 h	48 h	72 h
pWPI-FOLR1-SKOV 3 (μg/ml)							
1.8	12	8.86 ± 0.69	17.43 ± 0.91	30.29 ± 0.84	16.54 ± 2.58	24.84 ± 2.69	32.28 ± 2.97
3.6	12	18.52 ± 0.97	50.63 ± 1.31	64.28 ± 1.45	19.50 ± 2.71	50.08 ± 3.85	65.68 ± 4.03
7.2	12	32.24 ± 1.13	67.69 ± 1.24	79.38 ± 2.01	35.28 ± 3.59	71.44 ± 4.51	79.17 ± 4.58
pWPI-SKOV3 (μg/ml)							
1.8	12	6.54 ± 0.45	9.73 ± 0.67	15.79 ± 0.32	10.08 ± 2.19	14.89 ± 2.58	15.32 ± 2.61
3.6	12	12.89 ± 0.73	27.24 ± 0.85	38.21 ± 0.89	12.39 ± 2.06	27.61 ± 2.77	39.83 ± 3.19
7.2	12	19.26 ± 0.89	56.21 ± 1.04	69.77 ± 1.43	24.19 ± 2.78	58.80 ± 4.11	65.63 ± 3.91
SKOV3 (μg/ml)							
1.8	12	5.67 ± 0.48	8.35 ± 0.61	14.6 ± 0.45	9.27 ± 1.86	14.36 ± 2.56	17.72 ± 2.67
3.6	12	13.95 ± 0.56	24.59 ± 0.78	41.48 ± 0.75	11.27 ± 1.98	23.58 ± 2.49	40.64 ± 3.28
7.2	12	16.25 ± 0.67	57.73 ± 1.18	67.42 ± 1.51	23.62 ± 3.12	54.87 ± 4.10	68.64 ± 4.35

### Cell cycle analysis of cells treated with cisplatin

**Fig. 7:**
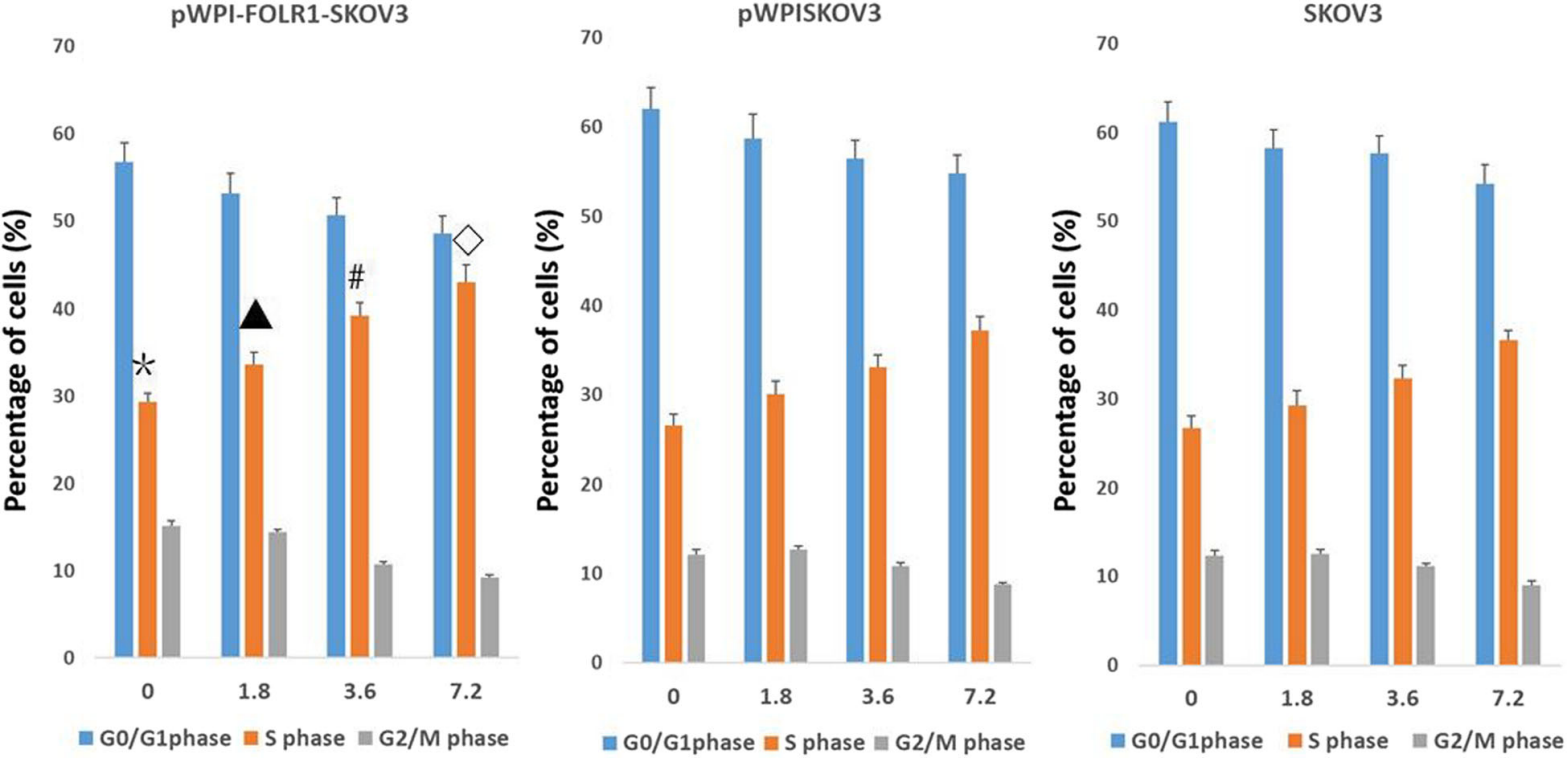
Cell cycle analysis of each of the three groups of cells treated with different concentrations of cisplatin (%, ±s). *,*P* < 0.05,compared with other two groups treated with cisplatin (0 μg/ml);▲*P* < 0.05,compared with other two groups treated with cisplatin (1.8 μg/ml);#,*P* < 0.05,compared with other two groups treated with cisplatin (3.6 μg/ml);⃟,*P* < 0.05,compared with other two groups treated with cisplatin (7.2 μg/ml)

Flow cytometry showed that the proportion of pWPI-FOLR1-SKOV3 cells in S phase (no cisplatin treatment) was significantly higher than the proportion in the other two control groups (*P* < 0.05). For all three groups, treatment with cisplatin (1.8, 3.6, and 7.2 μg/ml), increased the percentage of cells in S phase in a dose-dependent manner (*P* < 0.05). Under the same conditions, the percentage of cells in S phase in the pWPI-FOLR1-SKOV3 group was highest compared to the other two groups (*P* < 0.05) (Fig. 7).

### Concentration of residual cisplatin in cells detected by high performance liquid chromatography (HPLC)

**Fig. 8:**
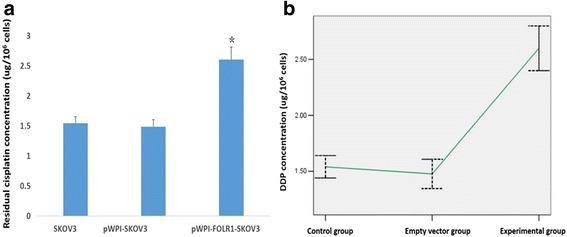
Residual cisplatin concentration in three groups treated by the same reference concentration after 48 h (**a**).*,*P* < 0.05,compared with the two control groups. Curve of residual cisplatin concentration in three groups treated by the same reference concentration after 48 h (**b**). Control group: SKOV3,Empty vector group: pWPI-SKO3,Experimental group: pWPI-FOLR1-SKOV3

The IC50 value in the pWPI-FOLR1-SKOV3 group was used as the reference concentration of cisplatin, and the same concentration of cisplatin was added to the three groups of cells, respectively. After 48 h of maturing they were used to detect the concentration of residual cispaltin in cells by HPLC (data not shown). Compared with standard curve, residual cisplatin concentration of the three groups were 1.543 ± 0.109 μg/10^6^cells,1.487 ± 0.115 μg/10^6^cells and 2.604 ± 0.205 μg/10^6^cells,respectively. The pWPI-FOLR1-SKOV3 cells displayed the highest concentration compared to the other two groups (*P* < 0.05). There was no significant difference between the two control groups (*P* > 0.05). The specific results are shown in Fig. 8.

## Discussion

In our previous study, we determined that FOLR1 was highly expressed in 160 ovarian tissue samples. This finding was consistent with another publications [7–9]. We find that FOLR1 is particularly high in cases of ovarian cancer, which suggests that FOLR1 may be useful as a clinical diagnostic marker for the disease. Yuan et al. [10] showed that expression of FOLR1 in ovarian cancer was significantly higher than in either breast cancer or malignant mesothelioma. CA125 has been routinely used as a serum biomarker of ovarian cancer. However, it has proven to be a poor diagnostic indicator of sensitivity and specificity for early stage disease [11]. Thus, additional biomarkers are needed. Combined detection of CA125 and FOLR1 may be useful for the early diagnosis of ovarian cancer [12]. Such combination could also improve specificity and treatment response prediction [13].

Ovarian tumors are typically treated with cytoreductive surgery and platinum-based chemotherapy. However, long-term efficacy is limited. Yakirevich et al. [14] found that only 75% to 80% of epithelial ovarian cancers respond to chemotherapy, while the rest display primary resistance. Eventually, all patients develop chemotherapy drug resistance, which contributes to a low five-year survival rate and poor prognosis. We found that expression of FOLR1 is significantly reduced in drug-resistant ovarian tumors compared to drug-sensitive tumors. These findings are consistent with the finding that cisplatin-resistant cells display decreased levels of folate binding protein [6]. Decreased expression of FOLR1 in ovarian cancer cells is significantly associated with drug resistance. Thus, this may represent a mechanism of multidrug resistance in the disease. Improvement of drug response has many potential benefits, including improving patient survival. We find that overexpression of FOLR1 in SKOV3 cells changes many of their biological properties, including cell cycle progression and apoptosis. SKOV3 cells overexpressing FOLR1 were the most sensitive to cisplatin treatment (IC_50_ = 3.60 μg/ml). These cells also displayed the most growth inhibition following treatment. Our data show that high expression of FOLR1 in ovarian cancer cells increases sensitivity to cisplatin. We hypothesize that FOLR1 may promote ovarian cancer cell growth by transporting folic acid; it may also influence the response to cisplatin. However, more work is needed to determine the mechanism responsible for this effect. In the late 1990s, Gibb et al. [15] found that cisplatin induces apoptosis of ovarian cancer cells. Cisplatin is currently used as first-line chemotherapy for ovarian cancer treatment. Chemoresistance is associated with apoptosis and cell cycle changes. Resistance of ovarian cancer cells to chemotherapy-induced apoptosis is a primary reason for treatment failure [16, 17]. Cisplatin is induced by the endogenous mitochondrial pathway of apoptosis in ovarian cancer [18]. Here, we show that increased expression of FOLR1 increases sensitivity of ovarian cancer cells to cisplatin. We hypothesize that changes in FOLR1 expression and folate metabolism directly or indirectly contribute to cisplatin-induced apoptosis in ovarian cancer and influence cisplatin sensitivity. A main therapeutic goal is to limit multidrug resistance and improve the clinical efficacy of chemotherapy. This, in turn, would likely improve patient prognosis and survival.

Here, we find that, in the absence of cisplatin treatment, the proportion of cells overexpressing FOLR1 in S phase is significantly increased compared to the two control groups. One group reported that FOLR1 expression in ovarian cancer negatively correlated with the loss of the potential tumor suppressor gene caveolin [19]. Here, we find that treatment of SKOV3 cells with cisplatin resulted in S phase arrest. Those with FOLR1 overexpression showed the most dramatic increase in S-phase fraction following cisplatin treatment, consistent with the fact that this group was most sensitive to the chemotherapy. This is the first description of its kind, as there are no other publications describing a link between this pathway and folate metabolism.

In summary, we find that FOLR1 is highly expressed in ovarian cancer but is reduced following multidrug resistance. FOLR1 may be a useful biomarker for ovarian cancer, and it may be useful as a therapeutic application to improve sensitivity to cisplatin treatment [20].

## Conclusions

In summary, we find that FOLR1 is highly expressed in ovarian cancer but is reduced following multidrug resistance. FOLR1 may be a useful biomarker for ovarian cancer, and it may be useful as a therapeutic application to improve sensitivity to cisplatin treatment [20].

## Methods

### Detection of FOLR1 protein in ovarian tissue

All ovarian tissues were lysed, and protein samples were run by SDS-PAGE. Proteins were then transferred to PVDF membranes. Membranes were blocked for one hour at room temperature in PBS containing 5% milk/0.1% Tween. Membranes were then washed in PBS-Tween (0.1%) and incubated overnight with FOLR1 goat anti-human polyclonal antibody and goat anti-human GAPDH polyclonal antibody in PBS containing 5% milk at 4 °C. The next day, membranes were washed with PBS-Tween and incubated for two hours at room temperature with infrared fluorescent dye-labeled donkey anti-goat secondary antibody in PBS containing 5% milk. Blots were washed with PBS-Tween, and proteins were visualized using chemiluminescence reagents. The infrared fluorescence Odyssey imaging system was used to scan and analyze gray values and to calculate the relative expression of FOLR1.

### Effect of cisplatin on SKOV3 cells

Full-length FOLR1 was PCR-amplified from pOTB7. Primer sequences contained EcoRI and XhoI sites. The amplified product was digested, purified, dephosphorylated, and then ligated into the pWPI vector to generate the recombinant plasmid pWPI-FOLR1. This recombinant plasmid was then transformed into *E. coli*, isolated, and sequenced. pWPI-FOLR1 was packaged with pCMV-dR8.74 and pMD2.G to produce lentivirus, which was used to infect SKOV3 cells. Flow cytometry using green fluorescence was performed to sort pWPI-FOLR1-SKOV3 positive cells. Control cells infected with lentivirus containing empty pWPI vector (pWPI-SKOV3) were generate in a similar manner. Untransfected SKOV3 cells were used as a blank control (SKOV3). Overexpression of FOLR1 was confirmed at the RNA and protein level by RT-PCR and Western blot, respectively.

### MTT assay following cisplatin treatment

Cells were cultured for 24 h and then treated with increasing concentrations of cisplatin (0.5, 1, 2, 4, 8, 16, 32, and 64 μg/ml); treatment was maintained for 48 h. The growth inhibition rate of each sample was calculated. Half maximal inhibitory concentration (IC_50_) software was used to obtain the IC_50_ of cisplatin in each of the three groups of cells. Concentration was normalized based on the IC_50_ of pWPI-FOLR1-SKOV3 cells treated with cisplatin. Concentrations of the three groups were 0.5 × IC_50_, 1 × IC_50_, and 2 × IC_50_, and cells were cultured for 24 - 72 h. Values for each well were calculated, and the inhibition rate of cell growth was determined.

### Flow cytometry to measure apoptosis and cell cycle progression following cisplatin treatment

Cells were cultured with increasing concentrations of cisplatin and incubated for 24, 48, and 72 h. PE-annexin V and 7-AAD (1 μl each) were mixed and added to cells. This was incubated for 15 min at room temperature in the dark. Next, 400 μl of ice-cold binding buffer was added and mixed gently before the cell preparations were examined by flow cytometry. Flow cytometry was then used to analyze the rate of apoptosis. For cell cycle analysis, cells were treated with cisplatin for 48 h. 1 μl RNA enzyme and 10 μl Triton X-100 were added to cells at room temperature for 30 min. Next, 1 μl PI was added and incubated at 4 **°**C for 30 min in the dark. Finally, flow cytometry was performed.

### Statistical analysis

All experiments were performed at least three times, and statistical analysis was performed using SPSS13.0 software (SPSS Inc., Chicago, USA). The values were expressed as mean ± SD. ANOVA was used for comparisons made between more than two groups. Significance was set at *P* < 0.05. Dunnett’s post hoc test was used to analyze multiple comparisons. *P* values of less than 0.05 (*P* < 0.05) were considered to be statistically significant.

## Abbreviations

7-AAD: 7-amino-actinomycin
ANOVA: The analysis of variance
CA125: Cancer antigen 125
CR: Complete response
DDP: Cisplatin
DMEM: Dulbecco’s modified eagle’s medium
DMSO: Dimethyl sulfoxide
DNA: Deoxyribonucleic acid
EOC: Epithelial ovarian cancer
FIOG: International Federation of Gynaecology and Obstetrics
FOLR1: Folate binding protein
GAPDH: Glyceraldehyde 3-phosphate dehydrogenase
GPI: Glycosyl phosphatidylinositol
HPLC: High-performance liquid chromatography
IC_50_: Half maximal inhibitory concentration
MDR: Multi-drug resistance
mRNA: messenger RNA
MTT: 3-(4,5-dimethyl-2-thiazolyl)-2,5-diphenyl-2-H-tetrazolium bromide
PD: Progressive disease
PI: Propidium iodide
PR: Partial response
PVDF: Polyvinylidene fluoride
RNA: Ribonucleic acid
RT-PCR: Reverse transcription polymerase chain reaction
SD: Stable disease
SDS-PAGE: Sodium dodecyl sulphate-polyacrylamide gel electrophoresis
